# “Let’s Just Wait Until She’s Born”: Temporal Factors That Shape Decision-Making for Prenatal Genomic Sequencing Amongst Families Underrepresented in Genomic Research

**DOI:** 10.3389/fgene.2022.882703

**Published:** 2022-05-20

**Authors:** Julia E. H. Brown, Astrid N. Zamora, Simon Outram, Teresa N. Sparks, Billie R. Lianoglou, Matthew Norstad, Nuriye N. Sahin Hodoglugil, Mary E. Norton, Sara L. Ackerman

**Affiliations:** ^1^ Program in Bioethics, University of California, San Francisco (UCSF), San Francisco, CA, United States; ^2^ Institute for Health and Aging, UCSF, San Francisco, CA, United States; ^3^ Department of Social and Behavioral Sciences, UCSF, San Francisco, CA, United States; ^4^ Department of Obstetrics, Gynecology, and Reproductive Sciences, UCSF, San Francisco, CA, United States; ^5^ Institute for Human Genetics, UCSF, San Francisco, CA, United States; ^6^ Center for Maternal Fetal Precision Medicine, UCSF, San Francisco, CA, United States; ^7^ Department of Surgery, UCSF, San Francisco, CA, United States; ^8^ Eli and Edythe Broad Center of Regeneration Medicine and Stem Cell Research, UCSF, San Francisco, CA, United States

**Keywords:** ELSI, prenatal exome sequencing, temporality, equity, genomic medicine

## Abstract

Genomic sequencing has been increasingly utilized for prenatal diagnosis in recent years and this trend is likely to continue. However, decision-making for parents in the prenatal period is particularly fraught, and prenatal sequencing would significantly expand the complexity of managing health risk information, reproductive options, and healthcare access. This qualitative study investigates decision-making processes amongst parents who enrolled or declined to enroll in the prenatal arm of the California-based Program in Prenatal and Pediatric Genome Sequencing (P3EGS), a study in the Clinical Sequencing Evidence-Generating Research (CSER) consortium that offered whole exome sequencing for fetal anomalies with a focus on underrepresented groups in genomic research. Drawing on the views of 18 prenatal families who agreed to be interviewed after enrolling (*n* = 15) or declining to enroll (*n* = 3) in P3EGS, we observed that the timing of sequencing, coupled with unique considerations around experiences of time during pregnancy and prenatal testing, intersect with structural supports beyond the clinic to produce preferences for and against prenatal sequencing and to contain the threat of unwelcome, uncertain knowledge. Particularly for those without structural supports, finding out consequential information may be more palatable after the birth, when the first stage of the uncertain future has been revealed. Future research should examine the role of temporality in decision-making around prenatal genomic sequencing across diverse population cohorts, in order to observe more precisely the role that structural barriers play in patient preferences.

## Introduction

After a 22-week ultrasound of their fetus, Erica and David were told that the sonographer “couldn’t find her brain–that was the first thing, and when that happens sometimes the baby dies in the stomach before she is born,” Erica recalled. There was also an apparent heart defect. Their first worry was that their baby would not carry to term. They were invited and agreed to participate in our study, through which detailed genomic sequencing was performed for their fetus, to improve medical understanding of the multiple structural differences observed. The sequencing took 4 weeks to complete. This is a fast turnaround time for sequencing more generally; however in the prenatal context and for Erica and David it meant that by the time the results were returned, “the time to terminate the pregnancy was over.” Besides, explained Erica, “I felt bad at that time because I could already feel her moving.” The sequencing identified a pathogenic variant in a gene associated with a brain malformation called Dandy-Walker Syndrome, as well as developmental delay, heart defects, scoliosis and additional complications.

David reflected: “everything can change all of a sudden: Suddenly you look at life and in a moment the panorama completely changes. It is not easy; that’s why many people make drastic decisions, like ending the pregnancy, or not doing the tests because people prefer not to know anything because it is not easy. Science is very advanced and that is nice, but sometimes with those news not everyone is prepared.”

On the one hand, there was personal reassurance: finding a non-inherited genetic cause meant that it was “nobody’s fault, it is something they don’t know why it happened—that is the purpose of the tests, to clarify” (David). On the other hand, there was uncertainty: “what I’m worried about now is the heart surgery, because they told us it would be done when she is born (…) maybe in 2 months or maybe sooner, and if she’s going to need medications too (…) we don’t know about her brain, if it is minimum or if it will be a lot. We’ll see” (Erica). “It is unpredictable, that’s the word. We can’t say anything because we don’t know. Nobody knows. We know about her heart; we know that she has a cyst in the brain and that’s our greatest concern. But regarding the rest, we don’t know” (David).

Until the birth, nothing felt actionable yet. “We are not in this process yet,” Erica said, “we don’t know what we will have to deal with, we only have to wait. The only thing is that I think these tests should been done earlier. As I said, before you start feeling the baby moving inside."

The experience of time and decision-making during the prenatal period is fraught. There is a future-oriented tension between prenatal diagnostics—indicating a prognosis for the postnatal experience—and the lived experience of what the fetus already is as a prenatal entity (Völkle and Wettmann 2021). Both the visualizations of the fetus *via* ultrasound and the lived experience of fetal movement, as Erica explained, affirm the fetus as a present, living entity. Any concerning information revealed by structural anomalies on the (approximate) 20-week ultrasound can introduce “sudden” uncertainty about what is to come. The decision to undergo further testing from this point must thus be seen within the context of existing uncertainty, introduced by the ultrasound. For Erica and David, a heart defect was identified *via* ultrasound and, even though it was not clinically part of the genomic sequencing, they had conflated both concerns together. Decision-making is not always contingent on genetic findings; personal beliefs and expectations vary (Richardson and Ormond 2018), while decisions about termination (when available) are yet to rest on genomic sequencing results (Kalynchuk et al., 2015). Erica and David had passed their personal threshold for the time by which they might have terminated the pregnancy. They felt that they now could only wait for their future baby’s needs to be revealed after birth. The genomic sequencing result offered some further explanation but ultimately not enough to act upon.

Prenatal genomic sequencing seeks to improve prenatal diagnosis by understanding the reasons for, and potential additional implications of, structural anomalies detected on routine prenatal ultrasound that are not detectable by standard chromosomal microarray or karyotype testing (Lord et al., 2019; Petrovski et al., 2019). Whole exome sequencing evaluates the protein-coding regions of the genome and identifies disease-causing genetic variants. For fetuses with undiagnosed structural abnormalities and otherwise ‘normal’ microarray results, exome sequencing can provide diagnostic information in as little as 6 percent and as many as 80 percent of cases (Best et al., 2018). This much variation is due to contextual factors, including the number of structural abnormalities observed and whether or not both parents in addition to the fetus can be sequenced (Mellis et al., 2018). While contested in its utility for whole population reproductive healthcare (ISPD et al., 2018), genomic sequencing is increasingly utilized in situations where a fetal structural anomaly is detected (Best et al., 2018). This trend is likely to continue (Fleck and Leslie, 2022).

There are, however, several logistical, experiential and equity challenges of prenatal genomic sequencing that warrant attention. First, timeliness is a huge barrier: turnaround time for sequencing results needs to be faster than in postnatal settings, where the window of potential action or treatment is wider (Kalynchuk et al., 2015). The late gestational age that anomalies are picked up when disorders are detected *via* imaging and the frequent need for another referral for a diagnostic procedure, which takes time, along with the current protocol requiring a microarray first, all compound the added delay of the sequencing process itself—not to mention the stakes now imposed by abortion bans pertaining to gestational age categories. Second, further research is needed to understand how genetic diseases manifest in a fetus and what the implications are of specific genetic variants identified *in utero*. Third, and related to the need for more timely sequencing, there is an impetus to provide access to adequate genetic counselling that takes into account the absence of clear phenotypes and prenatal reference data (Jelin and Neeta, 2018). Economic value for prenatal (and postnatal) interventions, encompassing a pipeline of testing *and* treatments, would need to be raised to meet health payer coverage (Trosman et al., 2020). Finally, patient acceptability of genomic testing across diverse population groups is not equal (Gutierrez and Hailu, 2021).

It is critical to capture the views of families who are underrepresented in genomic research. Populations who do not participate in genomic research remain underrepresented in two central ways. First, families are underrepresented in genomic databases, which thus use limited genetic ancestry information to drive the advancement of diagnostics and precision therapeutics (Sirugo et al., 2019). Individuals who are classified by European descent make up 81 per cent of genomic databases (Popejoy and Fullerton 2016). Second, underrepresented ancestry groups may also experience compounding structural inequalities, including systemic racism (Smith et al., 2016; Lee et al., 2019). For instance, Erica and David were from an ethno-racial minority group, they relied on government health insurance, and English was their second language. They had accessed further prenatal tests through participation in our study. Prenatal sequencing in the United States in its current form is available through exclusive and unequal access at the same time as adding another burden of ‘choice’ within prenatal care (Yurkiewicz et al., 2014). Yet, as Erica and David illuminated, revelations of uncertainty in prognosis do not just concern one intervention over another. Revelations of uncertainty begin with an ultrasound, before the sequencing option. Being able to pursue a prenatal diagnosis *via* any means is therefore associated with a burden of choice that gives rise to complex, time-pressured decision-making processes—particularly for groups who are underrepresented in genomic research.

This paper investigates how expectant parents from underrepresented groups in genomic research decide whether or not to pursue prenatal genomic sequencing—and the potential ongoing uncertainty it entails—in the context of limited opportunities for action before birth. As Erica and David described, pursuing prenatal genomic sequencing after a concerning ultrasound involves an “unpredictable” experiential process, despite the efforts of researchers and genetic counsellors to prepare expectant parents. Understandably, “people prefer not to know anything because it is not easy”—to hold uncertain information at the same time as, for the pregnant person, feeling at a visceral level the life of their unborn baby “moving inside” them, legitimizing hope and parental care.

Previous research on the temporality of pregnancy and prenatal tests suggests that there are experiential clashes between the linear stages of time informed by ultrasounds and biometric measurements, which give rise to gestational age and birth due dates, and how pregnant persons experience time during pregnancy as more precarious and ultimately negotiable in terms of when the birth takes place—as the first opportunity for post-test actionability (Sänger 2015). It has also been suggested that parents undergoing exome sequencing of their fetus can over-estimate the potential for answers and are likely to be unprepared for the increased uncertainty presented by results (Chandler et al., 2018; Richardson and Ormond 2018). Further, there are limitations to clinical capacities to manage uncertain results for those who pursue genetic testing prior to exome sequencing. Chromosomal microarray–identifying aneuploidy and structural changes in chromosomes that are typically not detectable by standard karyotype tests–places great demand on genetic counselors and obstetricians to account for diagnostic/prognostic uncertainty in a time-sensitive way. Thus microarray results have been described as sometimes imposing “toxic knowledge”: knowledge that is not wanted and makes expectant parents feel anxious throughout the remainder of pregnancy, in fear of what might be to come (Bernhardt et al., 2013). Through attention to decision-making processes, temporality, and structural supports, our study ultimately considers the extent to which prenatal genomic sequencing produces more “toxic knowledge”—on top of ultrasound findings—for underrepresented groups in particular, and how experiences of time intersect with decision-making about that potential knowledge.

## Methods

### Participants

The University of California, San Francisco (UCSF) initiated the California-based Program in Prenatal and Pediatric Genome Sequencing (P3EGS) in 2017. This was one of six NIH-funded sites in the Clinical Sequencing Evidence-Generating Research (CSER) consortium, investigating both prenatal and pediatric contexts. The main goal of P3EGS was to investigate the clinical and personal utility of exome sequencing, with a focus on underrepresented populations in genomic research. In the prenatal arm, utility applies to prenatal exome sequencing in situations of fetal structural anomalies. Most participating P3EGS families would otherwise be unable to access exome sequencing for their fetus’ or child’s suspected genetic condition, often due to a reliance on Medicaid/Medi-Cal coverage. Compared to the pediatric arm of the study where most parents (81.9%) relied on Medicaid/Med-Cal, expectant parents in the prenatal arm were predominantly privately insured (73.3%) and had higher incomes.

In addition to selecting participants to maximize inclusion of underrepresented groups, in the case of ongoing pregnancies inclusion also required participant willingness to undergo an amniocentesis first, for which a negative result was reported. We therefore had a selective subgroup of underrepresented populations who, with prior access to prenatal testing, were already dealing with an emotional toll of an anomalous pregnancy at baseline. Participants also underwent genetic counselling to help prepare them for the possibility of more uncertainty with the sequencing findings.

### Data Collection

Our analytic sample included 18 families who agreed to be interviewed after either enrolling (*n* = 15) or declining to enroll (*n* = 3) in the prenatal arm of P3EGS. Parents of probands (the affected fetus) were invited to participate in semi-structured interviews. The interview sampling strategy aimed to reflect the greater P3EGS cohort, while capturing the specific populations’ experiences (underrepresented families). The semi-structured interview guide was developed by the Ethical, Legal and Social Implications (ELSI) research team and included a wide range of topics, as well as specific questions related to the pursuit of prenatal genomic sequencing. Interviews were conducted either at the family’s home, over the phone, or *via* videoconference. Each interview had a duration of between 30 and 60 min. All interviews were conducted by three members of the ELSI research team with training in ethnographic data collection. Most interviews were conducted in English or Spanish, the latter of which were transcribed and translated to English for coding and analysis.

### Data Analysis

Qualitative analysis of interview transcripts involved thematic coding (Boyatzis 1998; Braun and Clarke 2006). An inductive approach was implemented whereby emerging patterns and themes were determined a *posteriori*. Data were analyzed using a pre-discussed set of qualitative codes. Codes were developed following what was being learned through initial observations and interviews. The ELSI research team iteratively conducted the process of coding and generating themes to increase the reliability of the iterative analysis. Themes were summarized to gain insight and provide an overall picture of the reasoning for each family’s pursuit for prenatal genomic sequencing.

## Results

Below we describe how temporal factors shaped decision-making amongst 18 families who agreed to be interviewed after either enrolling (*n* = 14), enrolling and not receiving results (*n* = 1) or declining to enroll (*n* = 3). Building on Erica and David’s case, we cite interviews from eight of these families, including five participants who enrolled and the three who declined to enroll. For families quoted in this paper, we use pseudonyms to balance the protection of participant identity and data integrity (Saunders et al., 2015). Table 1 reports on select demographics for all families, including whether families are considered underrepresented in genomic research by ethno-racial status (yes or no) and whether families were enrolled in government insurance (Medicaid/Medi-Cal) (yes or no). Given that most people giving birth in California are enrolled in Medicaid/Medi-Cal, our study sample indicates disparities in access and a selection bias towards those who could access private health insurance for prenatal care.

**TABLE 1:** Participant demographics.

Interviewee(s) names or participant ID	Under represented in genomic research	Medicaid/Medi-Cal	Enrollment status	Sequencing result	Pregnancy status at time of interview
Erica and David	Y	Y	Participant	Positive, *de novo*	Ongoing
Eva	Y	N	Participant	Inconclusive	Ongoing
Jane	N	N	Declined enrollment		Ongoing
Mei	Y	N	Participant	Inconclusive	Terminated (prior to participation)
Melissa	Y	N/A	Declined enrollment		Ongoing
Rachel & Jay	Y	N	Participant	Positive, *de novo*	Ongoing
Susan	N	N	Declined enrollment		Ongoing
Vina & Jim	Y	N	Participant	Negative	Ongoing
Fam 309	N	N	Participant	Positive, *de novo*	Ongoing
Fam 11	Y	Y	Participant	Negative	Terminated
Fam 348	N	N	Participant	Negative	Terminated
Fam 370	Y	N	Participant	Negative	Ongoing
Fam 398	Y	N	Participant	Positive	Ongoing
Fam 41	Y	Y	Participant	Inconclusive	Terminated
Fam 442	N	N	Declined results		Terminated
Fam 596	Y	Y	Participant	Inconclusive, *de novo*	Ongoing
Fam 195	N	N	Participant	Positive, *de novo*	Ongoing
Fam 86	Y	N	Participant	Positive, *de novo*	Ongoing

We observed that decisions to participate in prenatal genomic sequencing are guided by time availability, social supports, and confidence in being able to plan for an uncertain future. These factors may be influenced by broader structural and socioeconomic conditions, which along with temporality ought to be better accounted for in considerations of the potential benefits and harms of prenatal sequencing and how these are distributed. We have categorized our results under two key findings: 1) Decision-making takes time and support beyond what the clinic can provide; and 2) In the absence of timeliness and actionability, expectant parents keep the future open for as long as possible.

### Decision-Making Takes Time and Support Beyond What the Clinic can Provide

Making the decision about participation in prenatal sequencing takes time and personal assurance. The time it took participants to process relevant information may extend outside of when clinical advice is received, for several reasons. First, sorting through information in the clinical setting naturally invites more attention to medical concerns. Jane who declined to participate explained:

I feel like being in the hospital setting and always kind of under that pressure or I feel like there’s always this analysis going on about looking for potential risks—everything is very like risk-focused … I want to feel empowered.

Even expectant parents who, on the contrary, felt empowered while at the clinic to participate still sometimes changed their minds after leaving the clinic. For instance, after having blood drawn “with the intention of participating,” Susan and her partner walked back on their decision:

We talked to (the genetic counsellor) about it and … she was saying that they would use that to … narrow down, you know, [fetus’] sequence. So, I thought about that when we were talking to her, but then I thought more on it later, over the weekend, after we had talked about it. And we were like, you know what, let’s just not … you can only do what you feel is best in that moment.

Second, for those who participated in our study, decision-making was often based on feeling more able to think clearly after leaving the clinical setting. Rachel explained, “it was a lot of information. She [the genetic counselor] gave us a lot of information that we … we hear everything here and then we just, in the car, right, we start processing it.” Her partner, Jay, also described:

Afterwards we thought about the implications. We didn’t necessarily think about them all in the moment. I don’t think in the conversation itself we necessarily said “do we actually—you know, what does this come back to? You know, what does that mean and how do we react to it?” That part is, you know, it took some time to kind of process that and think that through and get to that stage of conversation. It wasn’t much longer. I think maybe on the car ride home.

Others felt overwhelmed regardless of the clinical or personal setting they were in. Melissa, who declined to participate, recalled:

I kind of just stayed quiet and they gave me a call and I was like, “You know what, let’s just, you know, let’s not do this. I’m just really scared, just terrified.” … me being stubborn and selfish and just scared, I was like, “Okay, I just don’t want to know until she’s born.” … I just had so much going on, like my mind was, like, going blank.

Patients struggled to process the fetal anomalies, and the option of genetic testing on top of that was often just too much.

Mei, who participated in our study after terminating her pregnancy, described feeling similarly overwhelmed in processing information pertaining to the sequencing results. While inconclusive, there was an indication of “MEHMO,” characterized by severe intellectual disability, epileptic seizures, hypogenitalism, microcephaly and obesity. Mei described that the order of delivery of information may have interfered with her ability to process what she was hearing:

(the genetic counsellor) kind of went straight into “This mutation is called this thing.” And it was so technical that I, A) could not really follow, even though I’m medically trained, I could not really follow … I was just surprised that there was a result at all. So, I wasn’t really following the details and I just found it to be very technical for like a very long time. And hard to figure out, like, what does this mean? … I actually wished that I had gotten a synth“esis from a physician first, to set the stage for “This is where you are about to hear,” and then go into, “Okay, there’s this thing called MEHMO. There’s this many identified pathogenic mutations.” You know,” you have a non-pathogenic, or a not identified variant,” you know, like, all the details would’ve followed better after the high level synthesis, or just some kind of mental preparation. Like, you know, “Out of the spectrum of outcomes, “here’s where you are and now let me tell you the details” … some kind of guiding statement would've helped.

Mei had made the decision to terminate based on both a follow-up fetal MRI, which “confirmed the diagnosis of agenesis of the corpus callosum,” and the seeking a second opinion. She explained that participating in P3EGS came “quite some time after that—after we had the termination … And, I honestly wasn’t expecting [the study] to find anything; I just thought, ‘I support research and so I don’t mind going through the process’.” Upon receiving the results for which she felt completely unprepared for, she felt the need for personal space to process this new information:

I had assumed that, just from the correspondence, there seemed to be no urgency, no rush. There was, I think (the genetic counselor) was even surprised that I was at work and not like somewhere, you know, more private … I don't think I was mentally prepared to be in a quiet place, you know, with some privacy, to really soak it in.

Finding “a quiet place” meant finding a supportive place—that would either affirm or challenge initial views. For Jane, who had “decided during the interview session” not to participate, it was in talking through the decision with her friends, who had “said, ‘Good for you’ … and just talking with other mothers, it seems like, you know, a lot of moms have felt like it has caused a lot of unnecessary stress in the pregnancy.” There was a sense of solidarity and trust in others about her reasoning.

That said, sometimes final decision-making differed between partners. Melissa, who had declined genomic sequencing for their fetus due to feeling overwhelmed at the time, later agreed to have genetic testing on herself, however her partner refused. The test result brought her personal relief: “with me, there was no trait or anything that could have been passed down to (Proband) … that was a big thing for me … I felt so guilty for the longest time until they told me, “No, it is not your fault …” It was just like eating me alive. It was horrible.” Nonetheless, with this new knowledge about herself came the challenge of how to manage her family’s expectations:

it’s just so hard to even like explain to my family now … my family has been driving me nuts, just asking all these questions … my family is just typical Mexican. They’re like, “Oh, she’ll get over it, she’ll get over it, she’ll get over it.” You know, that’s just them. So, I get frustrated when they ask me questions.

Finding a sense of ownership over the genomic sequencing experience takes time, which pregnancy cannot easily afford. Moreover, agreeing to participate in fetal sequencing can mean tempting a future that parents are not yet ready to accept.

### In the Absence of Timeliness and Actionability, Expectant Parents Keep the Future Open for as Long as Possible

Expectant parents face a tension between appreciating the as-yet-known health status of their fetus and using information to prepare for the birth of their baby. As described in reference to Erica and David, obtaining more information while being unable to act until a baby is born can be anxiety-inducing because there are still many unknowns: “we are not in this process yet … we only have to wait (until the birth).” Melissa explicitly stated about her decision to decline:

I just didn’t want to know like something horrible was going to happen to her, just like know this could happen when she’s born. It was just more like—then when she’s born, if this happens, it’s not more of a shock … It’s like, “let’s just wait until she’s born, just to know when she’s born.” Not to, like, know beforehand. It’s not a very pleasant feeling to know something is there.

After their daughter was born, Melissa had felt more inclined to have genetic testing not only for her own personal reassurance, but also because “now that her heart’s working perfectly, she’s looking good, it is like, ‘let’s just know, you know, now that she’s here.’ And if we could catch something now, that would be great … Now that she’s here, yes, definitely, so we could just catch something before it is too late.” It was only from birth onwards that the feeling of control over the future began to take hold.

Fear of finding out information before being able to act on it also took the form of declining to participate due to fears further down the line regarding secondary consequences of what might be disclosed:

If we did discover that we had some kind of preexisting condition that might be adult onset, like do we want to live in fear or anxiety about this like our entire lives? … we just wanted to live and not really be thinking about all those things … it was just opening up a can of worms and where would it end, where would all of this testing end? … what do we do with all of this information? … I’ve already had a few ultrasounds where they had different information about my due date. And, so, I don’t really like feeling all this anxiety.

While information about adult-onset conditions in a fetus was distinct and optional information, to be disclosed only if participants wanted, these expectant parents retained concern that there were no limits on the genetic information disclosed. Nonetheless, their decision-making seemed to involve having a greater sense of confidence in how knowledge can be put out of harm’s way when it is not relevant to present circumstances.

Some expectant parents asserted that prenatal genomic sequencing “provides us [with] even more information and being just educated … one less thing that we have to worry about” (Vina), or that “it gives us choices, and where knowledge is better than not having nothing” (Vina’s partner, Jim). Yet, as Erica described, this was perhaps dependent on how participants subjectively viewed the timeliness of the tests in regard to their personal (as well as the legal) thresholds for termination options.

Eva, who was not considering termination and decided to participate in P3EGS after a longer period of dealing with uncertainty having accessed a 12-week ultrasound, which revealed a potential heart anomaly, reflected:

It was kind of amazing to find out like at 12 weeks that they recognize, “hey, there’s something wrong,” you know, “something doesn't look right with the heart.” And to know that early on … it’s not what you want to hear but it’s beneficial in the big picture, in the sense that you can kind of prepare … what if we didn’t … and we just went along thinking “everything’s great, everything’s fine,” and then it’s not until he’s born that we’re—you know, and it would just completely derail us. Whereas it gave us a lot more time to kind of mentally prepare and figure things out as we went along, [rather] than just getting hit really hard at the end.

This contrasts sharply with Erica and David’s experiences, who, after receiving an abnormal ultrasound reading at 22 weeks and genomic sequencing results at 26 weeks, felt like the time to act had already passed. Again, selecting the appropriate social support can help buffer against fears of the future. In navigating the uncertainty of what was to come, David commented that “in life we have a person who … he wishes to share things with. It is nice because when I tell my mother, she will not say to me, ‘it is because of this or because of that.’ No, she tells me, ‘It is in God’s hands. He will know what do to’.” David had not shared the information with his siblings because “sometimes you prefer not to worry people, especially when it is such a hard situation. So far, we have it under control and I prefer not to tell them.” Only after baby’s birth would the couple be confronted with familial reactions that they did not feel ready for.

Other parents who declined genomic sequencing were able to, for the duration of pregnancy at least, feel confident in the hopeful information given through prior prenatal testing, rather than tempting more information through sequencing:

I just don’t feel like there’s any reason to do further testing … the other test that we did came out normal. There wasn’t anything unusual … I was comfortable with the results, where I felt like I didn't really need to delve deeper into the exome sequencing study … I think I just wanted to really trust my intuition and … I feel like the baby is healthy and I don’t really want to go through further testing when all the test results that we’ve gotten back so far have been normal. (Jane)

Accepting prenatal genomic sequencing could also mean a burden to think even further down the line into the future. Jane, who explained that they were confident in the information already provided through ultrasound, thus declining sequencing, summarized the full extent of their concerns: “I didn’t want to live in fear and anxiety based on the results of the study, and I also didn’t want it to affect my child or our potential ability to get insurance.” Beyond questions of healthcare coverage (which, contrary to some confusion, is protected under discrimination laws), there were legitime concerns about future life insurance coverage and the multitude of uncertain future implications for the future child. Susan, who had decided to pull out at the last minute, elaborated:

We didn’t feel that—specifically, that getting the information from the exome mapping for our daughter, [fetus], was going to change how we felt about her treatment or any decisions that we made about her treatment in the short term … it wasn't really going to affect us directly, but if it would be something that would be helpful for other families in the future … But then (the genetic counsellor) kind of reiterated, well, “there is information that you may find that may become helpful for you” … it was clear that there was potential for that … I remember it registering in my brain somewhere … but … we were still going to proceed and participate. And then the conversation with my husband about like, “oh, well, maybe this could negatively impact her in the future,” you know, that was kind of the deciding factor for us to pull out.

Expectant parents can view the actionability of genetic testing results in pregnancy as limited by the lack of available fetal treatments. Especially if the results are uncertain, “it is not enough to make decisions on, it is not definitive but it is also not as reassuring,” explained Mei.

For those seeking longer-term reassurance, prenatal genomic sequencing threatened to disrupt shorter-term confidence. Out of the 316 prenatal families who participated in exome sequencing in our study, nine families chose not to receive their results after the test results were ready. At least two of these families (that could be reached) said explicitly that they declined because they did not wish to learn the results. In these situations, there was nothing to act upon with the current pregnancy: one ended in termination and the other ended in miscarriage. The couple who terminated their pregnancy were from an underrepresented ethno-racial group who also relied on Medicaid. Further, 7/9 of the families that did not receive results relied on Medicaid and a respective 7/9 were identified as underrepresented ethno-racial group.

Expectant parents who chose to terminate their pregnancy appeared to feel that the information, while overwhelming to process at the time, became relevant upon further reflection when starting to plan for future pregnancies. Mei, who terminated her pregnancy before receiving the result, explained how she wished she had “screenshot” the information sent through after the consult rather than having to wait for it to be mailed out, because “the height of the issue was the couple of days after,” when her and her partner, who had conceived the terminated pregnancy *via* IVF, were deciding—a few days after the sequencing result came through—whether to transfer one of their remaining embryos already available to do another IVF cycle and seek a preimplantation genetic diagnosis. In seeking more information, “if I had pushed this any further, we would have been past the decision point of which I’m going to transfer.” They drew on information they recalled from return of results to “read more about it, understand more, and then to make a decision,” which was to transfer a female embryo “to be safe” because the inconclusive result was an “X-linked” condition.

Parents also often erred on the side of hope, giving possible undesirable outcomes the benefit of doubt, when making decisions to pursue current or future pregnancies. Mei rationalized that the variant of unknown significance was reported “because it is a variant in a gene that has some kind of brain effect, even though it is the same thing that our fetus actually had … it was more of a conservative reporting” and “even though we don’t know if it is a pathogenic or not pathogenic variant, I still suspect that probably not.”

## Discussion

This paper has demonstrated the importance of experiences of temporality in considerations about prenatal genomic sequencing—both the unique timing factors imposed by the prenatal period of clinical timeframes and the lived experiences of time pressures and structural barriers when faced with making decisions about sequencing and waiting for results. Prenatal testing has long produced a ‘tentative pregnancy’ (Rothman 1986). For expectant parents, decision-making around genomic sequencing may rest on the extent to which sequencing makes their pregnancy feel even more tentative at a time when women like Erica are already “feeling the baby moving inside.” Consistent with previous research on temporal experiences and conflicts imposed by prenatal testing (Sänger 2015), interviewees in our study described processing information and managing uncertainty in a non-linear way compared with clinical expectations. Declining prenatal genomic testing may help to suspend future uncertainties about outcomes, allowing parents to maximize control of the pregnancy. Critically, the greater ability to delay the possibilities of illness while a future baby remains *in utero* stands in contrast to newborn screening for genetic disease. In the contrasting case of newborn screening, parents of newborns are left to navigate the possibility of ‘illness in spite of symptoms or a diagnosis’ such that they become ‘patients-in-waiting,’ navigating between ‘an unremarkable state of “normalness” and “disease”’ that ultimately requires ‘patience’ until clear symptoms manifest (Timmermans and Buchbinder 2010: 417; 418). In the prenatal period, expectant parents are more likely to experience a sense of indeterminability and inactionability, which lends to more empowerment to make decisions on their own terms.

At a practical level, the search for more information becomes more productive when information is actionable. While prenatal interventions are available for some fetal anomalies and genetic diseases, such as *in utero* transfusions for inherited anemias, prenatal interventions at this time do not exist for most disease and are generally not curative. Thus, finding out consequential information may be more palatable either before pregnancy (*via* expanded parental carrier genetic testing, which many are not offered) or else after the birth when the first stage of the uncertain future has been revealed.

In the absence of prenatal treatments or the possibility of termination (depending on both patient and provider views and state regulations), parents may feel that nothing is actionable until the point of birth. Melissa had described declining genomic sequencing because she wanted to “just wait until she’s born … It is not a very pleasant feeling to know something is there,” without being able to know for sure or to do anything about it. For others, like Eva, participating in prenatal genomic sequencing provided “a lot more time to kind of mentally prepare and figure things out as we went along [rather] than just getting hit really hard at the end.” Eva, however, had begun the process of mental preparation at the 12-week ultrasound. Most parents eligible for prenatal genomic sequencing will not find out about structural anomalies until the 20-week ultrasound.

Prenatal genomic sequencing expands the orbit of managing health risk information, reproductive options, and systemic healthcare barriers introduced by earlier prenatal technologies. Racial disparities have long been a concern (Taylor et al., 2019), including in terms of access to prenatal genetic counseling (Christopher et al., 2022), a service that provides a critical opportunity for discussion about the level of uncertainty that might be acceptable to different expectant parents (Harris et al., 2018). Historically in California, declining earlier prenatal tests such as maternal serum alpha-fetoprotein (AFP), chorionic villus sampling (CVS) and amniocentesis was associated with racial and ethnic minority status and English language barriers (Kuppermann et al., 1996; Press and Browner 1998). Factors such as discomfort with and trust in clinical protocols and social rapport with clinicians, skepticism of statistical predictions, and religious beliefs were found to also shape declinations of amniocentesis, although these factors can be construed along social class lines as much as ethno-racial lines (Rapp 1998). For instance, acceptance of amniocentesis may be more likely amongst parents with higher education rather than ethnic or racial determinants per se (Saucier et al., 2005). In the case of today’s genomic sequencing, less is known about the dynamics of prenatal social barriers beyond logistical and access issues (Bernhardt et al., 2013). For pediatric patients with rare disease, social demographic variables such as limited healthcare access and English language barriers can exclude parents from support groups, lowering the perceived utility of genomic sequencing for parents (Halley et al., 2022). Our study suggests that for expectant parents who have undergone ultrasound and amniocentesis to now be considering genomic sequencing, there may also be issues around what structural supports are in place to deal with return of results should they imply that further healthcare and support will be needed post-birth. English speaking barriers may compound these needs. Having supports in place to manage the outcomes of genomic sequencing is critical, and this factor may become more pressing the further along in the pregnancy parents are.

In terms of decision-making about whether to participate in genomic sequencing in the first instance, there were numerous temporal and structural factors at play. Some expectant parents appeared less likely to get reassurance about their decision-making process from within the clinical setting. Amongst the minority who decide while in the clinic, they still sometimes changed their minds upon talking it through with family. Earlier research on why expectant parents decline maternal serum alpha-fetoprotein (AFP) suggests there is an association between taking more time to decide to decline AFP and being able to talk it over with family (Press and Browner 1998). We were unable to test this hypothesis specifically, however our findings point to the need for expectant parents to seek guidance beyond the clinic. Given previous ethnographic findings that underrepresented groups may be more likely to decline amniocentesis following consultation with family members (Rapp 1998), it is pertinent to consider how a sense independence from clinical input is either collective (family) or individual. Even if family members are present during clinical conversations, they might remain silent until returning to private spaces where they feel more empowered, and less encumbered by a lack of social relatability with clinicians, to express their concerns (Rapp 1998). Our finding that there can be a divergence between expectant parents in their decisions to pursue their own exome sequencing as additional information for the fetus (demonstrated in the case of Melissa) suggests that whereas family-influenced decision-making may have been historically colored by gendered roles (Rapp 1998), female-identifying expectant parents can assert more independence in their decision-making while still consulting family.

The tendency to seek one’s own information and social support in the prenatal period may depend on structural supports. This process may serve as a precursor to the ongoing “therapeutic odyssey” that parents face in the pediatric context, where genomic sequencing is only one part of a larger process that happens outside of the clinical setting (Childerhose et al., 2021). For other expectant parents, and perhaps more likely those with greater structural supports, they were more likely to feel confident in either the information presented in the clinical setting in the moment or to change their minds later after talking with their social supports. A previous Canadian study of decision-making about non-invasive prenatal testing (NIPT) to test for Down Syndrome observed that just over half of expectant parents envisaged being able to make a decision within the appointment where information was given about NIPT, with the rest preferring to take a few days to consider (Laberge et al., 2019). The study also found that previous knowledge (about Down Syndrome and NIPT) played little role in decision-making: expectant parents ‘do not necessarily need different types of information, but they simply need time to reflect on how to integrate this new knowledge into their decision-making process along with their values and preferences’ (Laberge et al., 2019). Parents in Laberge et al.‘s study were predominantly white, with access to universal prenatal healthcare. Our study suggests that, in practice, decision-making may be even less likely to happen within the same information session for expectant parents facing structural barriers.

The time taken to process information has important implications for informed consent. Amongst the larger P3EGS cohort, there was poor recollection of deciding whether or not to consent to broad data sharing of genomic information (Norstad et al., 2021). Previous research also suggests that, although expectant parents appreciate clinical support in their decision-making about prenatal whole exome sequencing, they would appreciate if the sequencing results were more timely, with more attention given to uncertainty, and with a preference for results to be repeated and delivered *via* multiple formats, as a way of ensuring more understanding of results (Quinlan-Jones et al., 2017). We have highlighted in this paper, however, that the delivery of information (and the timing of consent) is also complicated by the incongruence between the level of preparedness that might be expected from clinicians delivering information and the time, space and support that expectant parents may need beyond the clinical setting to sort through uncertain information and empower themselves in the decision-making process.

### Implications for Future Research

Future research should examine the role of temporality in decision-making around prenatal genomic sequencing across diverse population cohorts, to observe more precisely the role those structural barriers play in patient preferences. Returning to our question posed in the Introduction, of whether prenatal genomic sequencing may produce more “toxic knowledge” for expectant parents and clinicians to navigate (Bernhardt et al., 2013), our study has demonstrated that the experience of liminal time in the prenatal period, as well as social supports beyond the clinic, may help families to contain the threat of unwelcome, uncertain knowledge. As underrepresented groups in genomic research are also disadvantaged by having less access to social and structural supports that shape health (Smith et al., 2016), it is critical consider how clinical supports may be better harnessed to enable timely planning toward the future and acting on uncertain information.

Often underrecognized by both patients and providers is that identifying a prenatal diagnosis also allows for advance preparation for continuing pregnancies. For example, plans can be made for a fetus found to have an inborn error of metabolism to be delivered at a tertiary care institution, with a metabolic geneticist and availability of enzyme replacement therapy. The utility of such considerations merit further exploration with patients as they weigh decisions about whether to undergo genomic sequencing during pregnancy. Even in the absence of an intervention, there may be opportunities for more frequent monitoring and meeting with subspecialists to plan for delivery and to manage expectations. That said, for those who decline sequencing, it is important to give expectant parents space to run with the hope that they might have about an alternative future, which keeps them from tempting the unknown. Refusing more information can in some circumstances be preferable and empowering during what is already such a stressful time.

## Data Availability

The raw data supporting the conclusion of this article will be made available by the authors, without undue reservation.

## References

[B1] Bernhardt B. A. Soucier D. Hanson K. Savage M. S. Jackson L. Wapner R. J. (2013). Women's Experiences Receiving Abnormal Prenatal Chromosomal Microarray Testing Results. Genet. Med. 15 (2), 139–145. 10.1038/gim.2012.113 22955112 PMC3877835

[B2] Best S. Wou K. Vora N. Van der Veyver I. B. Wapner R. Chitty L. S. (2018). Promises, Pitfalls and Practicalities of Prenatal Whole Exome Sequencing. Prenat. Diagn 38 (1), 10–19. 10.1002/pd.5102 28654730 PMC5745303

[B3] Boyatzis R. E. (1998). *Transforming Qualitative Information: Thematic Analysis And Code Development*. Transforming Qualitative Information: Thematic Analysis and Code Development. Thousand Oaks, CA, US: Sage Publications, Inc.

[B4] Braun V. Clarke V. (2006). Using Thematic Analysis in Psychology. Qual. Res. Psychol. 3 (2), 77–101. 10.1191/1478088706qp063oa

[B5] Chandler N. Best S. Hayward J. Faravelli F. Mansour S. Kivuva E. (2018). Rapid Prenatal Diagnosis Using Targeted Exome Sequencing: A Cohort Study to Assess Feasibility and Potential Impact on Prenatal Counseling and Pregnancy Management. Genet. Med. 20 (11), 1430–1437. 10.1038/gim.2018.30 29595812

[B6] Childerhose J. E. Rich C. East K. M. Kelley W. V. Simmons S. Finnila C. R. (2021). The Therapeutic Odyssey: Positioning Genomic Sequencing in the Search for a Child’s Best Possible Life. AJOB Empir. Bioeth. 12 (3), 179–189. 10.1080/23294515.2021.1907475 33843487 PMC9922533

[B7] Christopher D. Fringuello M. Fought A. J. Bolt M. Micke K. Elfman H. (2022). Evaluating for Disparities in Prenatal Genetic Counseling. Am. J. Obstetrics Gynecol. MFM 4 (1), 100494. 10.1016/j.ajogmf.2021.100494 34583054

[B8] Fleck L. M. Francis L. (2022). Should Whole Genome Sequencing Be Publicly Funded for Everyone as a Matter of Healthcare Justice? Camb Q. Healthc. Ethics 31 (1), 5–15. 10.1017/s0963180121000426 35049449

[B9] Gutierrez A. M. Robinson J. O. Outram S. M. Smith H. S. Kraft S. A. Donohue K. E. (2021). Examining Access to Care in Clinical Genomic Research and Medicine: Experiences from the CSER Consortium. J. Clin. Transl. Sci. 5 (1), e193. 10.1017/cts.2021.855, 34888063 PMC8634302

[B10] Halley M. C. Young J. L. Fernandez L. Kohler J. N. Bernstein J. A. Wheeler M. T. Undiagnosed Diseases Network (2022). Perceived Utility and Disutility of Genomic Sequencing for Pediatric Patients: Perspectives from Parents with Diverse Sociodemographic Characteristics. Am. J. Med. Genet. Part A 188 (4), 1088–1101. 10.1002/ajmg.a.62619 34981646 PMC8923971

[B11] Harris S. Gilmore K. Hardisty E. Lyerly A. D. Vora N. L. (2018). Ethical and Counseling Challenges in Prenatal Exome Sequencing. Prenat. Diagn. 38 (12), 897–903. 10.1002/pd.5353 30171820 PMC6370459

[B12] ISPDS MFM PQF (2018). Joint Position Statement from the International Society for Prenatal Diagnosis (ISPD), the Society for Maternal Fetal Medicine (SMFM), and the Perinatal Quality Foundation (PQF) on the Use of Genome-wide Sequencing for Fetal Diagnosis. Prenat. Diagn 38 (1), 6–9. 10.1002/pd.5195 29315690

[B13] Jelin A. C. Vora N. (2018). Whole Exome Sequencing. Obstetrics Gynecol. Clin. N. Am. 45 (1), 69–81. 10.1016/j.ogc.2017.10.003 PMC5813701 29428287

[B14] Kalynchuk E. J. Althouse A. Parker L. S. Saller D. N. Jr Rajkovic A. (2015). Prenatal Whole-Exome Sequencing: Parental Attitudes. Prenat. Diagn 35 (10), 1030–1036. 10.1002/pd.4635 26151551

[B15] Kuppermann M. Gates E. Eugenewashington A. (1996). Racial-Ethnic Differences in Prenatal Diagnostic Test Use and Outcomes: Preferences, Socioeconomics, or Patient Knowledge? Obstetrics Gynecol. 87 (5), 675–682. 10.1016/0029-7844(96)00017-8 8677066

[B16] Laberge A.-M. Birko S. Lemoine M.-È. Le Clerc-Blain J. Haidar H. Affdal A. O. (2019). Canadian Pregnant Women's Preferences Regarding NIPT for Down Syndrome: The Information They Want, How They Want to Get it, and with Whom They Want to Discuss it. J. Obstetrics Gynaecol. Can. 41 (6), 782–791. 10.1016/j.jogc.2018.11.003 30738740

[B17] Lee S. S-J. Cho M. K. Kraft S. A. Varsava Nina. Gillespie Katie. Kelly E. (2019). I Don’t Want to Be Henrietta Lacks”: Diverse Patient Perspectives on Donating Biospecimens for Precision Medicine Research. Genet. Med. 21 (1), 107–113. 10.1038/s41436-018-0032-6 29887604 PMC6289900

[B18] Lord J. McMullan D. J. Eberhardt R. Y. Hamilton E. Rinck G. Hamilton S. J. (2019). Prenatal Exome Sequencing Analysis in Fetal Structural Anomalies Detected by Ultrasonography (PAGE): A Cohort Study. Lancet 393 (10173), 747–757. 10.1016/s0140-6736(18)31940-8 30712880 PMC6386638

[B19] Mellis R. Chandler N. Chitty L. S. (2018). Next-Generation Sequencing and the Impact on Prenatal Diagnosis. Expert Rev. Mol. Diagnostics 18 (8), 689–699. 10.1080/14737159.2018.1493924 29962246

[B20] Norstad M. Outram S. Brown J. E. Zamora A. N. Koenig B. A. Risch N. (2021). The Difficulties of Broad Data Sharing in Genomic Medicine: Empirical Evidence from Diverse Participants in Prenatal and Pediatric Clinical Genomics Research. Genet. Med. 10.1016/j.gim.2021.09.021 PMC12970631 34906477

[B21] Petrovski S. Aggarwal V. Giordano J. L. Stosic M. Wou K. Bier L. (2019). Whole-Exome Sequencing in the Evaluation of Fetal Structural Anomalies: A Prospective Cohort Study. Lancet 393 (10173), 758–767. 10.1016/s0140-6736(18)32042-7 30712878

[B22] Popejoy A. B. Fullerton S. M. (2016). Genomics Is Failing on Diversity. Nature 538, 7624161–7624164. 10.1038/538161a PMC5089703 27734877

[B23] Press N. Browner C. H. (1998). Characteristics of Women Who Refuse an Offer of Prenatal Diagnosis: Data from the California Maternal Serum Alpha Fetoprotein Blood Test Experience. Am. J. Med. Genet. 78 (5), 433–445. 10.1002/(sici)1096-8628(19980806)78:5<433::aid-ajmg8>3.0.co;2-m 9714010

[B24] Quinlan‐Jones Elizabeth. Hillman Sarah. C. Kilby Mark. D. Greenfield Sheila. M. (2017). Parental Experiences of Prenatal Whole Exome Sequencing (WES) in Cases of Ultrasound Diagnosed Fetal Structural Anomaly. Prenat. Diagn. 37 (12), 1225–1231. 29049852 10.1002/pd.5172

[B25] Rapp R. (1998). Refusing Prenatal Diagnosis: The Meanings of Bioscience in a Multicultural World. Sci. Technol. Hum. Values 23 (1), 45–70. 10.1177/016224399802300103 11660551

[B26] Richardson A. Ormond K. E. (2018). Ethical Considerations in Prenatal Testing: Genomic Testing and Medical Uncertainty. Seminars Fetal Neonatal Med. 23, 1–6. 10.1016/j.siny.2017.10.001 29033309

[B27] Rothman B. K. (1986). The Tentative Pregnancy: Prenatal Diagnosis and the Future of Motherhood. New York: Norton.

[B28] Sänger Eva. (2015). Obstetrical Care as a Matter of Time: Ultrasound Screening, Temporality and Prevention. Hist. Philosophy Life Sci. 37 (1), 105–120. 10.1007/s40656-014-0056-4 26013438

[B29] Saucier J. B. Johnston D. Wicklund C. A. Robbins-Furman P. Hecht J. T. Monga M. (2005). Racial-Ethnic Differences in Genetic Amniocentesis Uptake. J. Genet. Couns. 14 (3), 189–195. 10.1007/s10897-005-0641-5 15959650

[B30] Saunders B. Kitzinger J. Kitzinger C. (2015). Anonymising Interview Data: Challenges and Compromise in Practice. Qual. Res. 15 (5), 616–632. 10.1177/1468794114550439 26457066 PMC4582834

[B31] Sirugo G. Williams S. M. Tishkoff S. A. (2019). The Missing Diversity in Human Genetic Studies. Cell. 177 (1), 26–31. 10.1016/j.cell.2019.04.032 30901543 PMC7380073

[B32] Smith C. E. Fullerton S. M. Dookeran K. A. Hampel H. Tin A. Maruthur N. M. (2016). Using Genetic Technologies to Reduce, rather Than Widen, Health Disparities. Health Aff. 358, 1367–1373. 10.1377/hlthaff.2015.1476 PMC5100696 27503959

[B33] Taylor J. Novoa C Hamm K Phadke S. (2019). Eliminating Racial Disparities in Maternal and Infant Mortality: A Comprehensive Policy Blueprint. Center for American Progress.Available at: https://www.americanprogress.org/issues/women/reports/2019/05/02/469186/eliminating-racial-disparities-maternal-infant-mortality/ .

[B34] Timmermans S. Buchbinder M. (2010). Patients-in-Waiting. J. Health Soc. Behav. 51 (4), 408–423. 10.1177/0022146510386794 21131618

[B35] Trosman J. R. Weldon C. B. Slavotinek A. Norton M. E. Douglas M. P. Phillips K. A. (2020). Perspectives of US Private Payers on Insurance Coverage for Pediatric and Prenatal Exome Sequencing: Results of a Study from the Program in Prenatal and Pediatric Genomic Sequencing (P3EGS). Genet. Med. 22 (2), 283–291. 10.1038/s41436-019-0650-7 31501586 PMC7004856

[B36] Völkle Laura. Wettmann Nico. (2021). The Process of Pregnancy: Paradoxical Temporalities of Prenatal Entities. Hum. Stud. 44 (4), 595–614.

[B37] Yurkiewicz I. R. Korf L. S. Lehmann. L. S. (2014). Prenatal Whole-Genome Sequencing? Obstetrical Gynecol. Surv. 69 (4), 197–199. 10.1097/01.ogx.0000446907.09890.ff

